# Integrating fiber-optic seismic arrays into earthquake early warning systems with the dEPIC framework

**DOI:** 10.1038/s41598-025-30568-3

**Published:** 2025-12-10

**Authors:** Yuancong Gou, Ran N. Nof, Brian Pardini, Richard M. Allen

**Affiliations:** 1 Department of Earth & Planetary Science, University of California, Berkeley, 94720 USA; 2 Berkeley Seismological Laboratory, University of California, Berkeley, 94720 USA; 3 Geological Survey of Israel, Jerusalem, 95501 Israel

**Keywords:** Engineering, Natural hazards, Solid Earth sciences

## Abstract

Distributed Acoustic Sensing (DAS) can enhance earthquake early warning (EEW) by transforming existing fiber-optic cables into dense seismic arrays, including in offshore areas with sparse instrumentation. We present dEPIC (DAS–Earthquake Point-source Integrated Code), the first operational DAS-integrated EEW framework, deployed on a submarine cable in Monterey Bay, California, and designed to operate independently or jointly with ShakeAlert’s EPIC algorithm. dEPIC combines GPU-accelerated machine-learning phase picking, grid-search location, and empirical magnitude estimation, with real-time quality metrics to suppress unstable solutions. In event replays and continuous data tests, dEPIC detected both onshore and offshore earthquakes accurately with sub-second processing time. The modular, edge-computing design enables adaptation to future DAS deployments to improve existing EEW systems like ShakeAlert.

## Introduction

Earthquake Early Warning (EEW) systems are designed to rapidly detect and characterize earthquakes and issue timely alerts to regions expected to experience hazardous shaking^1–3^. The effectiveness of EEW systems is highly dependent on seismic networks; a spatially dense, low-latency, real-time network ensures timely and accurate warnings. Such networks have enabled the operation of successful EEW systems in many regions^4–12^.

Enhancing EEW capabilities from an observational standpoint often focuses on expanding sensor coverage in seismogenic regions, such as along active faults and subduction zones. Because most EEW algorithms require good azimuthal coverage and spatially proximal observations to rapidly and accurately locate earthquakes, challenges arise at the edges of traditional seismic networks^13,14^, especially in offshore regions where instrumentation is limited due to accessibility and the high cost of seafloor installation and maintenance^15,16^.

This limitation has motivated exploration of alternative sensing approaches, particularly those leveraging existing infrastructure^17,18^. Recent advances in fiber optic sensing technology have opened new opportunities for seismology by transforming existing fiber optic cables into dense seismic arrays^19–23^. Among these technologies, Distributed Acoustic Sensing (DAS) has emerged as one of the most mature and widely adopted methods, enabling a broad range of seismic applications^24,25^. For example, leveraging DAS on offshore telecommunications cables can supplement traditional seismic networks without the need for complex offshore deployments and extensive logistical support^26–37^. However, DAS data differ from conventional seismic observations due to their dense spatial sampling, linear array geometry, varying coupling conditions, large data volumes, and single-component axial strain measurements^38,39^. Recent studies have explored the feasibility of integrating DAS into Earthquake Early Warning (EEW) systems, showcasing its potential to augment existing capabilities^40–47^.

Operated by the U.S. Geological Survey (USGS), ShakeAlert is the official earthquake early warning system for the U.S. West Coast^48^. The USGS provides real-time earthquake information generated by ShakeAlert, while official delivery partners distribute public alerts through various communication channels^49,50^. EPIC (Earthquake Point-source Integrated Code) is ShakeAlert’s primary point-source EEW algorithm, designed to rapidly detect earthquakes using the first  4 s of P-wave arrivals from at least four stations. It estimates the earthquake hypocenter, origin time, and magnitude^48,51–53^. Here, we present dEPIC (DAS - Earthquake Point-source Integrated Code), a framework for the first operational DAS-integrated EEW algorithm. dEPIC can function independently or in conjunction with EPIC, to provide EEW solutions for the Monterey Bay region of California. Our system leverages the high spatial resolution of DAS using a machine learning–based phase picker, a grid-search event locator, and an empirical magnitude estimator. To address location errors caused by linear DAS geometry and phase-picking uncertainties in initial solutions, we developed new metrics to evaluate solution quality in real time. In addition, using EPIC solutions and its onshore traditional seismic stations allows us to further mitigate location ambiguities and identify distant events. The processing pipeline has been optimized with Graphics Processing Unit (GPU) acceleration to efficiently handle large data volumes, achieving sub-second processing times compatible with streaming data rates. With an edge-computing architecture, dEPIC can operate autonomously on a local server, issuing timely alerts for nearby events. Communication pathways between dEPIC and EPIC enable joint earthquake locations for associated detections. We present results applying dEPIC to both earthquake event data and continuous data. The modular design of dEPIC makes it adaptable to future DAS deployments aimed at enhancing EEW systems like ShakeAlert.

## Results

**Fig. 1:**
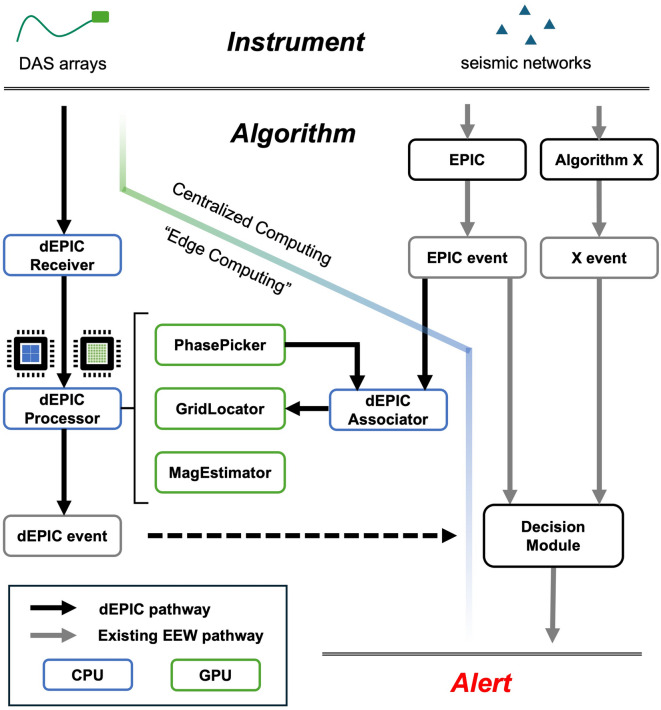
Overview of the dEPIC framework for earthquake early warning. The system integrates DAS arrays and conventional seismic networks. Real-time DAS data are processed using edge computing with GPU acceleration to detect earthquakes. When existing EEW algorithms such as EPIC detect an event, the dEPIC associator combines DAS-based detections with the seismic network detection for joint location. dEPIC events can then be passed to the decision module for potential alert issuance.

The dEPIC framework Fig. 1 consists of three main layers: data acquisition from the instrument, event characterization algorithms, and alert generation. The algorithm includes an edge-computing module that processes DAS data and an associator that listens for EPIC event messages. The dEPIC associator compares seismic phase arrivals observed on the DAS array with EPIC event information from the traditional network to determine whether both originate from the same earthquake event. If so, the data are combined for a joint location estimate. Location solutions are evaluated using the location metrics described in the Methods section, and solutions with a prominence score< 1.15 or a distribution score< 0.80 are rejected. Accepted events may be passed to ShakeAlert’s Decision Module for further evaluation before issuing an alert to the public. We present retrospective analyses (replays) of both recorded earthquake events and continuous data from the SeaFOAM (Seafloor Fiber-Optic Array in Monterey Bay) project , which turns a 52-km-long DAS array into a seismic array with 20.4 m gauge length and 5.1 m channel spacing^27^.

### Replay of individual events

We first applied dEPIC in simulated real-time processing of archived earthquake event data, selecting 21 events: all 13 onshore events with M>= 3.5 and 8 offshore events with M>= 2.0 in the Monterey Bay (within 100 km from the array) during the project. The list of earthquakes can be found in the Supplementary Materials (Figure S1). Each dataset contained 120 s of 200 Hz strain-rate waveforms, with the first P-wave arrival at the DAS array at around 30 s. The replayed data were processed together with contemporaneous EPIC messages, as obtained in real time. All replays were run using real-time configuration parameters, with a 1-second data update interval.

Of the 21 replayed events, 18 dEPIC detections were successfully associated with EPIC events, enabling joint location during the process. Three offshore events did not produce EPIC detections due to their small magnitude resulting in insufficient valid triggers onshore; however, dEPIC independently detected these events using DAS data alone.

**Fig. 2:**
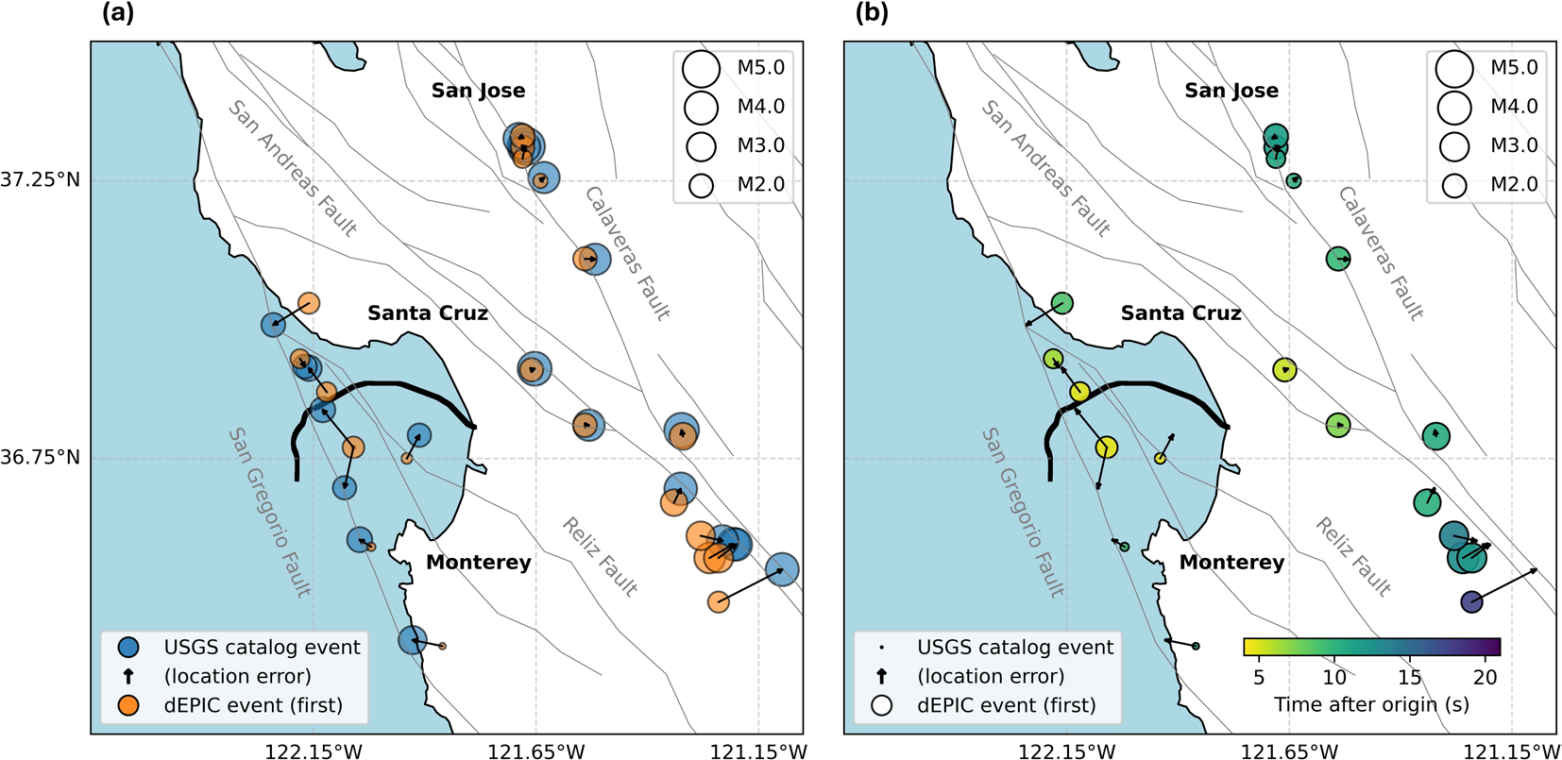
First accepted dEPIC solutions for replayed events. (**a**) Event locations and magnitudes from dEPIC (orange) compared with corresponding USGS catalog events (blue), including location errors shown as arrows proportional to the value. (**b**) Same events colored by time elapsed since the earthquake origin.

Figure 2 shows the earliest accepted solutions after applying location metric filtering (“first” solutions). In these cases, only the dEPIC P-wave magnitude estimates are shown to emphasize the rapid magnitude estimation capability; Fig. 2 (a) presents dEPIC first solutions with corresponding catalog event location and magnitude. Figure 2 (b) shows the time elapsed since the earthquake origin for each solution. While most events had reasonable initial locations, early magnitudes were underestimated because dEPIC uses the peak strain rate for magnitude estimation, and initial solutions often precede the amplitude peak. All onshore events have jointly solved initial locations, as regional seismic stations detected them before P-waves reached the DAS array. This helps dEPIC to correctly identify regional earthquakes because otherwise the stand-alone DAS array will have poorly constrained event solutions that may lead to false alerts. For 3 offshore events, located north of the cable and without EPIC detections, the locations relied solely on DAS data.

**Fig. 3:**
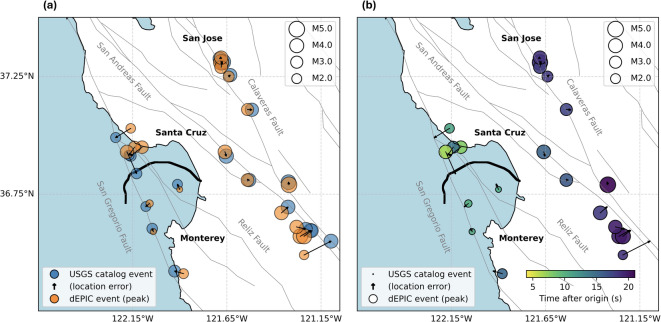
“Peak” accepted dEPIC solutions for replayed events. Same legend as in Fig. 2. The “peak” solution is defined as the time when the magnitude estimate first rose to within 0.1 magnitude units of the maximum estimated magnitude.

Figure 3 presents the “peak” solutions, defined as the time when the magnitude estimate first rose to within 0.1 magnitude units of the maximum estimated magnitude. This definition accounts for the gradual stabilization of magnitude estimates and is consistent with the magnitude update criteria in the ShakeAlert system^48^. Compared to the first solutions, these locations were slightly more accurate and stable, and magnitudes more closely matched catalog values due to more complete peak strain-rate data. Magnitude evolution plots for all replay events are provided in the Supplementary Materials (Figure S2).

### Replay of continuous data

**Fig. 4:**
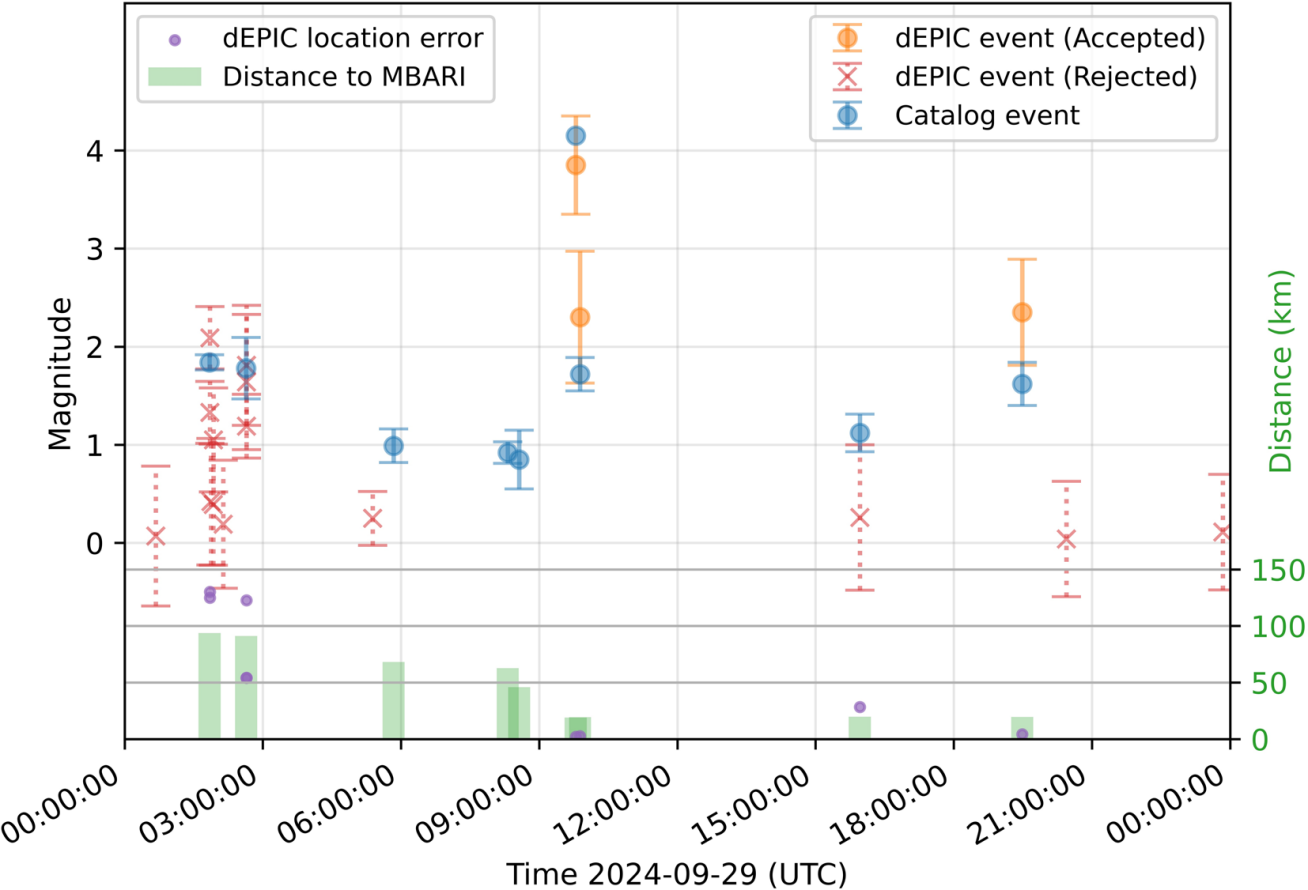
Full-day dEPIC replay results for 29 September 2024. Accepted (orange circles) and rejected (red crosses) dEPIC solutions with magnitude estimates and median absolute deviation values. Catalog events (blue circles) are shown with magnitude uncertainties. Green bars indicate event distances from the DAS array. Solutions with maximum magnitude estimates associated with catalog events are shown with location errors (purple dots).

**Fig. 5:**
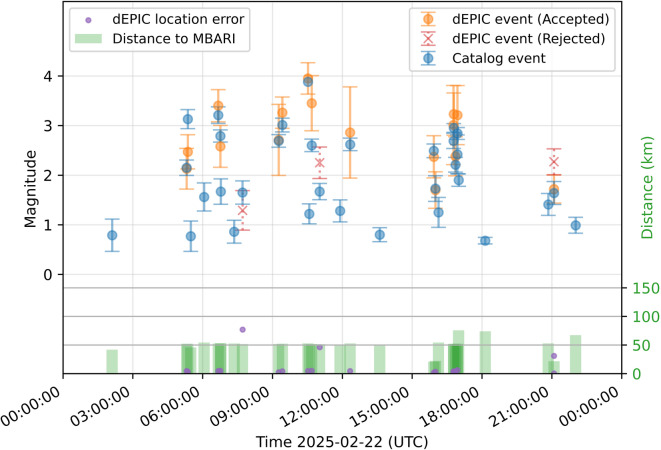
Full-day dEPIC replay results for 22 February 2025. Same legend as in Fig. 4.

We also tested dEPIC on continuous full-day data to assess its performance in routine detection and false-alarm suppression. Figure 4 and Fig. 5 show results for two days, illustrating detected events either accepted or rejected by the location metric filter. Catalog events within 100 km around the DAS array are shown for comparison.

On 2024-09-09 (Fig. 4), dEPIC successfully detected the M 4.2 mainshock on the San Andreas Fault and 2 out of 3 aftershocks ( 25 km from the DAS array). The smallest aftershock (M 1.1) was detected but rejected by the location filter, which also has large location error. Other rejected detections corresponded to small earthquakes more than 100 km away or to noise triggers.

On 2025-02-22 (Fig. 5), an ongoing earthquake sequence produced many events  50 km from the DAS array. All events with M>= 2.0 were detected and accepted with good location and magnitude estimates.  A few smaller events (M< 2.0) were detected but rejected correctly because they turned out to have large location errors. There are missed events due to temporal overlap with preceding events, or very small magnitude and greater distance.

### Real-time performance

We have installed a local server with an NVIDIA L4 GPU for the DAS array in Monterey Bay and run dEPIC in real time since July 28, 2025. Average data transmission latency measured is  0.5 seconds and the algorithms normally function with a 1-second data update rate. EPIC event messages are obtained from the ShakeAlert system, and dEPIC event messages are not used by the ShakeAlert operational system at this point. Between July 28 and August 12, 2025, the dEPIC system did not detect any event fulfilling the location metrics thresholds. A M3.1 event on July 31, 2025 04:28:00.480 (UTC), SE of Portola Valley, around 55 km from the DAS, was the largest magnitude offshore event during the real-time operation. Unfortunately the dEPIC associator was disconnected from EPIC due to technical issues during the event. The event message created by EPIC was not associated but dEPIC was functioning with only DAS data. The first solution for this event by dEPIC was 12 seconds after the catalog origin time, and none of the solutions passed the location metrics thresholds to create an alert message. Location errors for this event were 33 km and gradually reduced to 15 km. The initial magnitude estimate was 2.0 and reached a maximum magnitude of 3.6. This example demonstrates the advantage of using the location metrics to suppress poor solutions and to avoid false alerts. A replay shows that if the EPIC event was correctly associated, the first accepted solution at 12 seconds after origin time would have a location error of only 3.8 km and a peak magnitude estimation of 3.4. The technical problem was fixed and dEPIC has been running since Sep 10. As of October 20, there was only one onshore earthquake with a magnitude larger than 3 within 100 km of the array. dEPIC successfully detected this event (Figure S3).

## Discussion

The event replays demonstrate that dEPIC can deliver rapid and reliable earthquake locations and magnitude estimates through integration with existing EEW systems. For onshore events in the Monterey Bay region that are more than 20 km away from the DAS array, the integration helps dEPIC to generate accurate initial solutions. For some offshore events, dEPIC can provide early solutions based solely on DAS data followed by joint solutions with a smooth transition derived from the proposed location method.

**Fig. 6:**
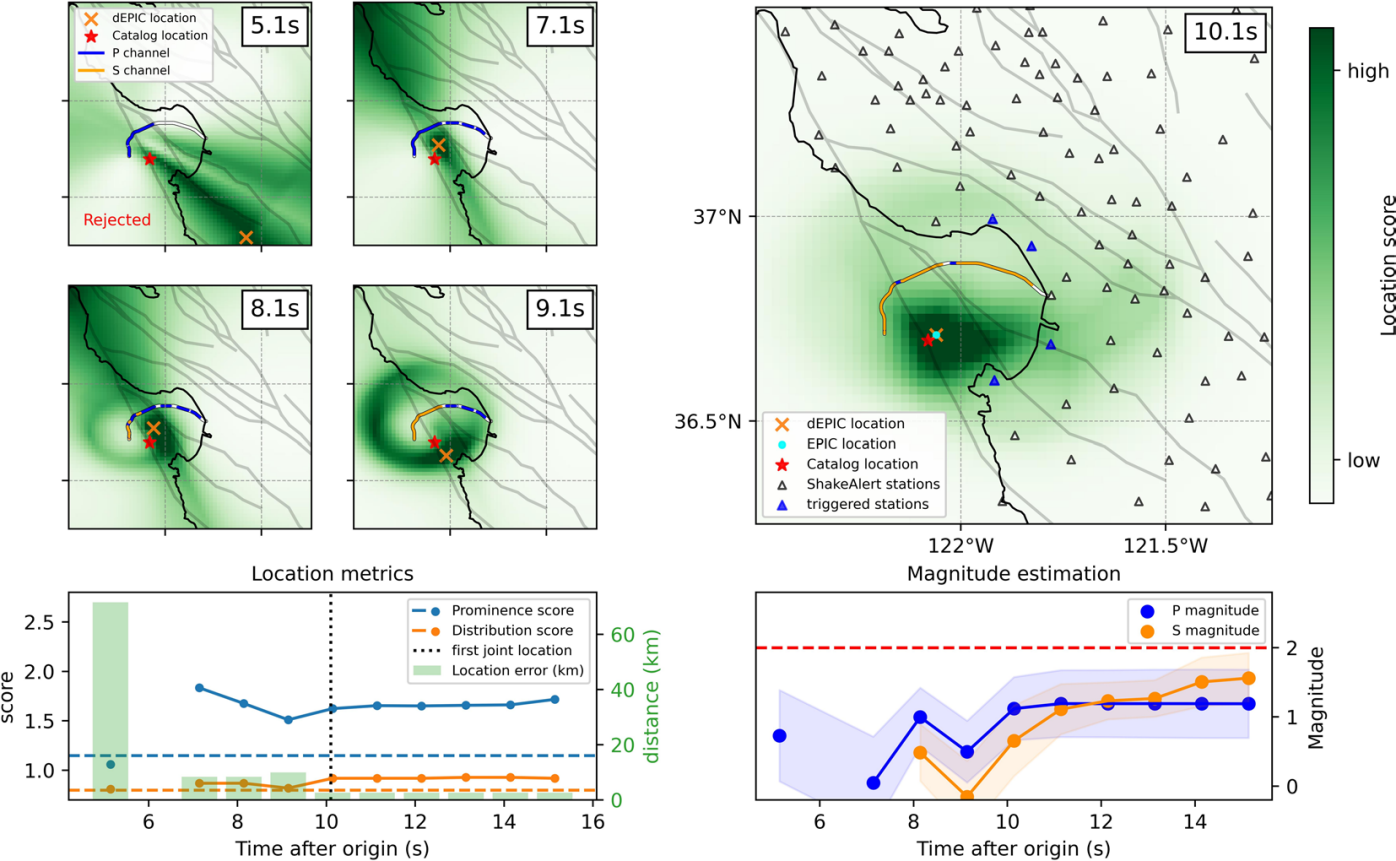
Example dEPIC solution snapshots for a M 2.0 offshore event (ID: nc75058341). (**a**) Location score snapshots from DAS data alone; (**b**) First joint location snapshot with seismic network data; (**c**) Evolution of location metrics and errors, including the first rejected solution; (**d**) Evolution of P- and S-wave magnitude estimates.

Figure 6 illustrates an offshore example, showing the algorithm’s decision process from an initial rejected poorly constrained solution, to early accepted detections based solely on DAS data,  and finally to a stable, jointly-solved location. As shown in Fig. 6 (a), the first solution is rejected due to low prominence and distribution scores. thereby avoiding a potentially large location error that could have produced a false event with an overestimated magnitude. Around 3 seconds of additional warning time is gained compared to the first EPIC solution. For some offshore events, dEPIC’s first accepted solutions precede the first EPIC-only locations by several seconds, depending on the location, consistent with the additional warning time estimated in our earlier study^40^ (Figure S4).

Although no large offshore earthquakes have yet occurred in the study region since implementation, the smaller events analyzed here suggest that dEPIC can provide accurate initial locations for such scenarios. A well-constrained location is essential for accurate magnitude estimation^54,55^. The likely magnitude error for larger offshore events in this region are more difficult to assess due to the lack of data. The estimated largest earthquake that happened on the offshore San Gregorio fault is around M7.8^56^. Local large magnitude events could cause amplitude saturation in DAS strain or strain-rate data, when the optical phase change between two laser pulses exceeds -π or ^57–60^. Using the theoretical maximum measurable strain rate given our DAS configuration and the empirical magnitude estimation equation we calibrated^40^, the DAS array on average should be sufficient to capture magnitudes up to 7.1 at 6.5 km distance using P waves, and magnitude 4.7 using S waves. This is higher than reported for the Long Valley DAS array^58^. The calibrated K term from our DAS data in Equation (1) is consistently smaller than in the original study^61^, which means that for the same magnitude and distance, our DAS data records lower peak strain-rate amplitude. This results in the increase of estimated magnitude saturation thresholds. The reason for the lower peak amplitude could be due to coupling conditions or local scattering. The detailed saturation magnitude at different hypocentral distances for all channels can be found in the Supplementary Materials (Figure S5). A recent study has also shown that the secondary phase (P-to-S converted phases) scales well with magnitude^45^. We do observe secondary phases in our data. It is worth exploring if the 3-second window we use for the peak-strain-rate calculation systematically includes secondary phases. From a practical EEW perspective, the point-source algorithm’s rapid detections are critical for issuing the first alerts for M 6+ events, which can be improved by algorithms designed for large magnitudes, such as FinDer^62,63^ (Finite-Fault Rupture Detector) and G-FAST^64^ (Geodetic First Approximation of Size and Time).

Future improvements could further shorten detection times. One key enhancement would be to enable joint location updates at the individual pick level, allowing DAS phase detections to be combined with EPIC station picks before an EPIC event message is issued. Since EPIC requires at least four stations to declare an event, this change could yield earlier joint solutions, though it introduces additional complexity in associating detections across systems. Another avenue is optimizing the current data update interval. While real-time processing is presently configured with a conservative 1 s update rate, actual computation times range from 0.2 s to 0.8 s. Reducing the processing time could enable shorter update intervals and faster detection. Several complementary approaches from other studies could also be explored. Mini-array processing strategies may help refine locations when DAS coverage is limited^44^, and combining dEPIC with Bayesian methods such as those introduced in bEPIC (Bayesian-EPIC) could incorporate prior seismicity information to improve early detections^15^. To address the risk of false triggers from teleseismic events, dEPIC could adopt EPIC’s existing filtering mechanisms or develop dedicated classifiers. Initial real-time tests showed that a distant Mw 8.8 event (July 29, 2025, Kamchatka earthquake) as well as its largest aftershock so far (September 18, 2025) did not trigger the system due to insufficient channel picks, likely because of differing frequency content between teleseismic phases and local seismic phases that the ML phase picker is trained on – an outcome that may be beneficial for EEW operation but not general phase detection. On October 10, 2025, two teleseismic events (M 7.6 - Drake Passage and M 7.4 - Santiago, Philippines) did not trigger the real-time system either (Figure S6). In another case, a distant Mw 4.5 Mendocino event generated a spurious small local solution, but it was rejected by the location quality metrics, illustrating the value of the location filter.

## Conclusion

We developed and deployed dEPIC, a real-time EEW framework that integrates DAS arrays with regional seismic networks through event association and joint-location methods. By combining a DAS array in Monterey Bay with the ShakeAlert network, dEPIC improves offshore coverage, delivers consistent solutions for onshore events and earlier solutions for some offshore events. We designed location metrics to reject poorly constrained detections. Tests with event and continuous data in Monterey Bay demonstrate that dEPIC can operate both independently and in coordination with ShakeAlert’s EPIC, enabling seamless transitions from DAS-only to joint solutions. Its modular, edge-computing design makes it readily adaptable to other DAS deployments, offering a practical approach to enhancing EEW. We aim to use dEPIC to integrate multiple DAS cables with ShakeAlert in the future, focusing on regions with limited station coverage, such as the Mendocino Triple Junction in Northern California.

## Methods

### Algorithm overview

The dEPIC framework consists of three core algorithmic modules–**PhasePicker**, **GridLocator**, and **MagEstimator**–designed to perform earthquake detection and characterization using DAS data. These modules operate under a point-source approximation, following a methodology similar to that used in EPIC. To meet the demands of EEW, they are optimized for real-time performance through parallelization and GPU acceleration. dEPIC is tailored for edge-computing environments and includes communication pathways to communicate with existing regional EEW systems, such as ShakeAlert in the U.S. west coast.

- **PhasePicker**: This module identifies seismic phases (P and S) in real time using a pre-trained convolution neural network, SeaFOAM PhaseNet-DAS^40,65,66^. The model was adapted through transfer learning to generalize from its original training on land-based DAS data to the offshore cable environment in Monterey Bay, California^40^. Each identified phase is assigned a phase score representing the model’s confidence, and only high-scoring picks above a fixed threshold are forwarded to the location module.
- **GridLocator**: This module determines earthquake locations by performing a grid search over a predefined geographic region near the DAS cable. It calculates the misfit between observed and expected arrival times based on a regional 1D velocity model. The module also listens for EPIC detection messages and attempts to associate nearby EPIC events with DAS phase picks. When an association is made, a joint location is performed using both DAS data and related seismic stations. To support this integration, we introduced a new weighting scheme and solution acceptance criteria, as described in the Joint Earthquake Location section in detail. The accepted location is then used as the preferred hypocenter for magnitude estimation.
- **MagEstimator**: This module estimates earthquake magnitude from the peak amplitude of DAS strain-rate waveforms using empirical scaling relationships calibrated for the local region^40^. It accounts for hypocentral distance and distinguishes between P- and S-wave–derived magnitudes. In Equation (1), $E_i$ is peak strain-rate after the P or S arrival, *M* is the magnitude, $D_i$ is hypocentral distance, and $K_i$ is the calibration term at the *i*-th channel that needs to be calibrated using local earthquake data. The magnitude coefficient *a* and distance coefficient *b* are 0.437 and 1.269 respectively for P-waves, 0.690 and 1.588 for S-waves^61^.
  1 $$\begin{aligned} log_{10}(E_i) = aM - blog_{10}(D_i) + K_i \end{aligned}$$
  The median value of magnitude estimation from all available channels is assigned as the event magnitude.

### dEPIC earthquake location method

#### Grid search

Event location is performed by evaluating arrival-time misfits over a spatial grid covering the region near the DAS cable. The rectangular grid search region centered around the DAS array is ($36.25^{\circ }$ N, $37.51^{\circ }$ N, $121.24^{\circ }$ W, $122.50^{\circ }$ W) with $0.02^{\circ }$ grid spacing. Phase arrivals (picks) from the PhasePicker module are filtered before the grid search. Invalid phase picks are excluded before computing the misfit. Specifically, we discard any pick that occurs at the latest sample of the time window, to ensure sufficient differential time among triggered channels for reliable location. Channels with S arrival times earlier than their corresponding P arrivals are also rejected.

To suppress spurious triggers, the grid search process is initiated only when more than 10% of the DAS channels have valid P-wave picks. At each grid point $\textbf{x}$ and time step *t*, a combined misfit is computed between the predicted and observed average relative P-arrival time, as well as the S–P differential time when available.

2 $$\begin{aligned} \mathscr {M}(\textbf{x}, t) = \sum _{i=1}^{N(t)} w_i \cdot \left| t_i^{\text {obs}} - t_i^{\text {pred}}(\textbf{x}) \right| , \end{aligned}$$

where $t_i^{\text {obs}}$ and $t_i^{\text {pred}}(\textbf{x})$ correspond to either a P arrival time or an S–P differential time, $w_i$ is the inverse of the phase score (a quality metric) for pick *i*, and *N*(*t*) is the number of valid picks at time *t*.

The misfit is transformed into a likelihood value using softmin normalization:

3 $$\begin{aligned} P(\textbf{x}, t) = \frac{\exp \left( -\mathscr {M}(\textbf{x}, t) \right) }{\sum _{\textbf{x}'} \exp \left( -\mathscr {M}(\textbf{x}', t) \right) }. \end{aligned}$$

To emphasize phase types observed on more channels, we assign separate coverage weights:

4 $$\begin{aligned} C_{\text {P}}(\textbf{x}, t) = \frac{N_{\text {P}}(\textbf{x}, t)}{N_{\text {total}}}, \quad C_{\text {S}-\text {P}}(\textbf{x}, t) = \frac{N_{\text {S}-\text {P}}(\textbf{x}, t)}{N_{\text {total}}}, \end{aligned}$$

where $N_{\text {P}}$ and $N_{\text {S}-\text {P}}$ denote the number of channels contributing P picks and S–P picks, respectively. $N_{\text {total}}$ is the total number of channels in the DAS array.

The final location score at each grid point is computed as:

5 $$\begin{aligned} S(\textbf{x}, t) = (C_{\text {P}}(\textbf{x}, t) -C_{\text {S}-\text {P}}(\textbf{x}, t)) \cdot P_{\text {P}}(\textbf{x}, t) + C_{\text {S}-\text {P}}(\textbf{x}, t) \cdot P_{\text {S}-\text {P}}(\textbf{x}, t). \end{aligned}$$

Since phase picking is performed over a finite sliding window, the number of contributing P picks may decrease as the wavefield passes. When this occurs:

6 $$\begin{aligned} N_{\text {P}}(\textbf{x}, t) < N_{\text {P}}(\textbf{x}, t - \Delta t), \end{aligned}$$

we update the score recursively to avoid generating inconsistent solutions :

7 $$\begin{aligned} S(\textbf{x}, t) \leftarrow S(\textbf{x}, t) \cdot S(\textbf{x}, t - \Delta t). \end{aligned}$$

Note that the location score is no longer restricted to the [0, 1] range but serves as a relative measure of plausibility, with higher values indicating increased confidence in the location estimate. We take the grid point with highest location score as the event epicenter. Finally, event origin time is determined based on the chosen event location and the median of the current arrival times.

#### Association and joint location

As shown in Fig. 1, we established a pathway to listen for EPIC detection messages, which contains event information (location, magnitude, origin time, associated triggered stations with triggering time etc.) determined by EPIC. To associate EPIC events with DAS phase detections, we estimate the expected arrival time of the EPIC event at the triggered channel with median arrival time using EPIC event location and origin time. If the absolute time difference between this predicted arrival time and the actual P trigger time on the median channel is below a configurable threshold (set to 5 seconds), the EPIC event is considered associated with the current DAS detections. Then the triggered EPIC stations within a certain range will be used in the joint location. Joint location follows the same method described in the previous section, but combines misfits from both DAS arrays and stations. The location score in the joint location case is defined as:

8 $$\begin{aligned} S_{\text {joint}}(\textbf{x}, t) = w_{\text {EPIC}} \cdot P_{\text {EPIC}}(\textbf{x}, t) + (C_{\text {P}}(\textbf{x}, t) -C_{\text {S}-\text {P}}(\textbf{x}, t)) \cdot P_{\text {P}}(\textbf{x}, t) + C_{\text {S}-\text {P}}(\textbf{x}, t) \cdot P_{\text {S}-\text {P}}(\textbf{x}, t), \end{aligned}$$

where $P_{\text {EPIC}}(\textbf{x}, t)$ is the softmin-normalized likelihood from EPIC station picks, and $P_{\text {P}}$, $P_{\text {S}-\text {P}}$ are the DAS-based components. The weights $C_{\text {P}}$ and $C_{\text {S}-\text {P}}$ are as defined previously.

The weight $w_{\text {EPIC}}$ allows tuning the relative influence of EPIC in the joint location process. We currently set $w_{\text {EPIC}} = 1$, which gives the EPIC misfit a higher weight. However, this value can be adjusted in future deployments to re-balance the contributions depending on network geometry or data quality.

#### Location evaluation metric

**Fig. 7:**
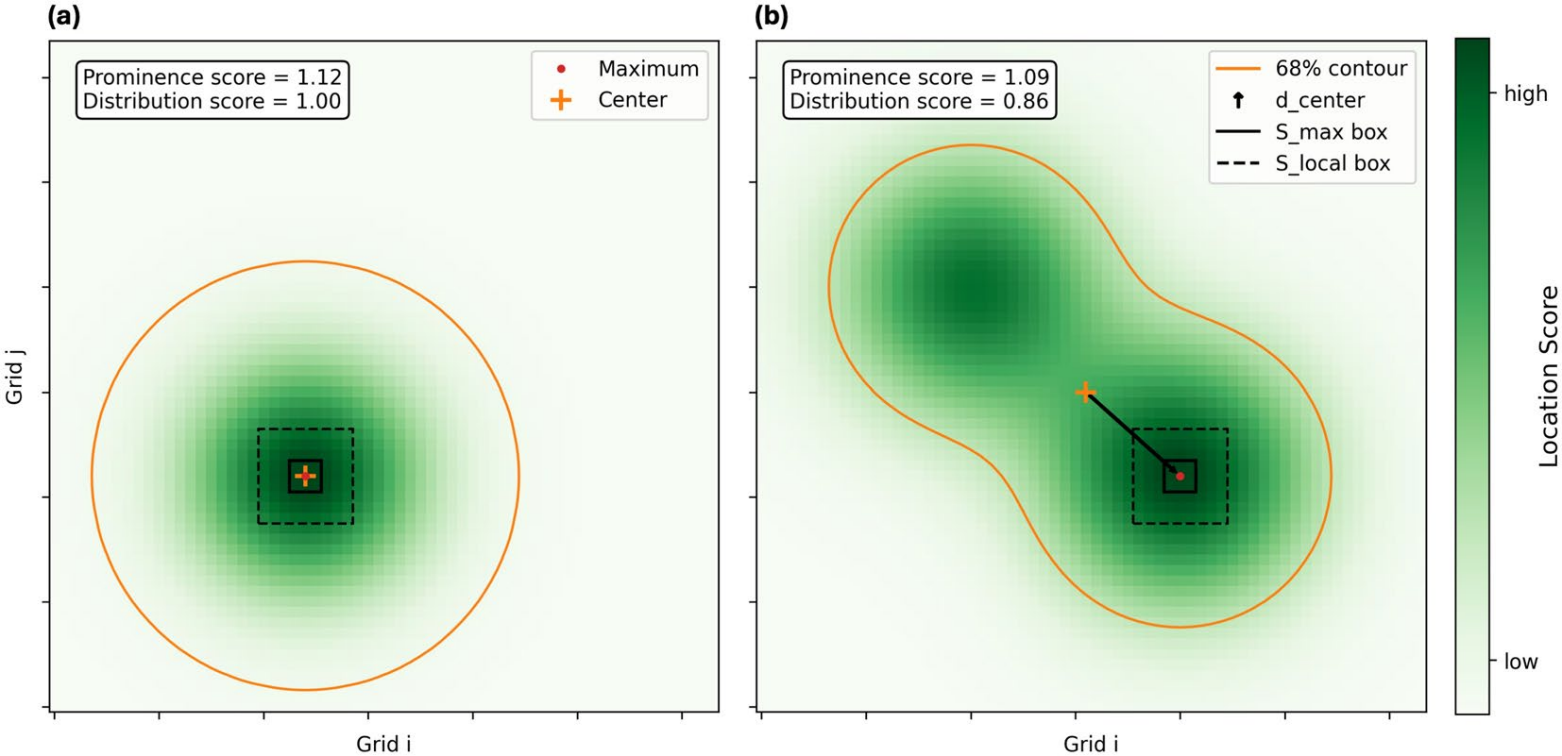
Illustration of location metrics. (a) Calculation of prominence and distribution scores for a well-constrained solution; (b) Same metrics for an ambiguous solution.

DAS arrays are often linear, and to ensure that only robust and well-constrained locations are forwarded to the alerting system, each candidate solution is evaluated using two complementary metrics derived from the spatial likelihood distribution $S(\textbf{x}, t)$ as shown in Fig. 7:

- **Prominence score**: This metric quantifies the sharpness of the highest-probability grid point relative to its local neighborhood. It is defined as:
  9 $$\begin{aligned} \text {prominence score} = \frac{\text {max}( S(\textbf{x}, t))}{\text {median}( S(\mathbf {x''}, t))}, \end{aligned}$$
  where $\mathbf {x''}$ is the outer edge of a 9$\times$9 box ($S_{\text {local}}$ box in Fig. 7) without the inner 3$\times$3 box ($S_{\text {max}}$ box in Fig. 7) centered at the peak. A higher score indicates a more sharply defined peak.
- **Distribution score**: This metric evaluates how well-centered the peak location is relative to the surrounding high-probability region. We first define the high-likelihood region as all grid points between the 68th and 100th percentiles of $S(\textbf{x}, t)$. We then compute the geometric center of this region and measure its distance to the maximum-likelihood point. The score is defined as:
  10 $$\begin{aligned} \text {distribution score} = 1 - \frac{d_{\text {center}}}{d_{\text {max}}}, \end{aligned}$$
  where $d_{\text {center}}$ is the Euclidean distance between the high-probability region center and the grid’s maximum point, and $d_{\text {max}}$ is the maximum possible distance on the grid (a constant). A higher score implies that the peak is well-aligned with the center of mass of the likely region, suggesting an azimuthally well-constrained solution.

Both metrics are evaluated for each location solution. If either score falls below a predefined threshold, the location solution is rejected. We have tested different thresholds of the two metrics to evaluate the trade-off between location accuracy and alert timeliness from the replay events. The current thresholds (prominence of 1.15 and distribution score of 0.80) balance the trade-off (Figure S7) and work well in real time.

### Algorithm optimization

To meet the requirements of a real-time EEW system, the data processing speed must exceed the data update rate. We stream 200 Hz DAS data for all 10300 channels from an OptaSense QuantX interrogator, with a latency around 0.53 seconds at the local server (Figure S8). We optimized SeaFOAM PhaseNet-DAS processing to eliminate unnecessary post-processing and deployed the grid search on the GPU to accelerate matrix operations. The data receiver, processor, and EPIC message receiver operate in parallel. We process 10-second-long sliding data windows. The current processing time varies depending on the number of picks detected by the SeaFOAM PhaseNet-DAS model. While the GPU inference for a fixed data window in PhaseNet-DAS is constant, pick extraction and filtering times vary, and CPU–GPU data transfer accounts for a significant portion of the total processing time. In contrast, grid search and magnitude estimation require relatively little time. At present, we can process one data window within 1 s (0.64 s on average with a standard deviation of 0.18 s from replay events), so the data update rate is set to 1-second interval.

## Supplementary Information

Supplementary Information.

## Data Availability

The codes and data needed to reproduce the figures can be found at https://doi.org/10.5281/zenodo.16861902. dEPIC-detected events were matched to events in the Advanced National Seismic System (ANSS) Comprehensive Earthquake Catalog (ComCat): https://earthquake.usgs.gov/data/comcat/ (last accessed August 2025). Figures were compiled using the matplotlib and Cartopy packages in Python. Fault data were obtained from U.S. Geological Survey and California Geological Survey, Quaternary fault and fold database for the United States at: https://www.usgs.gov/natural-hazards/earthquake-hazards/faults (last accessed August 2025).
