# Supplementary material for: Integrating fiber-optic seismic arrays into earthquake early warning systems with the dEPIC framework
Source: Sci Rep. 2025 Dec 10;16:960. doi: 10.1038/s41598-025-30568-3 (PMC12783127; doi:10.1038/s41598-025-30568-3)
Supplement: Supplementary file 1 — Supplementary Information. [file 41598_2025_30568_MOESM1_ESM.pdf]

Supplemental Material to ”Integrating Fiber-Optic Seismic Arrays into Earthquake Early Warning Systems with the dEPIC Framework”

Yuancong Gou, Ran N. Nof, Brian Pardini, Richard M. Allen

December 4, 2025

replay_events

ID	latitude	longitude	depth	magnitude	origin_time	description
**nc73799091**	37.312	-121.672	8.4	5.1	2022-10-25 18:42:02	M 5.1 - 15km ESE of Alum Rock, CA
**nc73866925**	36.801	-121.323	10.2	4.4	2023-04-04 22:23:17	M 4.4 - 1km NNW of Tres Pinos, CA
**nc75068161**	36.912	-121.653	6.9	4.2	2024-09-29 9:47:33	M 4.2 - 3 km of Aromas, CA
**nc73774300**	36.549	-121.097	1.6	4.0	2022-09-01 13:36:22	M 4.0 - 5km ENE of Pinnacles, CA
**nc75137161**	36.695	-121.325	4.7	3.9	2025-02-22 10:31:25	M 3.9 - 11 km SSW of Tres Pinos, CA
**nc73827436**	36.595	-121.206	7.7	3.9	2023-01-01 6:49:10	M 3.9 - 9km NW of Pinnacles, CA
**nc73856426**	36.594	-121.201	9.3	3.8	2023-03-07 14:33:12	M 3.8 - 9km NW of Pinnacles, CA
**nc75106461**	36.601	-121.231	7.6	3.6	2024-12-22 1:44:44	M 3.6 - 11 km NW of Pinnacles, CA
**nc73918291**	37.256	-121.632	5.9	3.6	2023-07-30 13:49:04	M 3.6 - 14 km N of Morgan Hill, CA
**nc73836266**	37.109	-121.517	5.7	3.6	2023-01-23 13:58:02	M 3.6 - 9km ENE of San Martin, CA
**nc73834041**	36.810	-121.531	5.3	3.6	2023-01-19 17:15:55	M 3.6 - 4km S of San Juan Bautista, CA
**nc73814941**	37.326	-121.688	8.2	3.6	2022-12-05 23:13:16	M 3.6 - 13km ESE of Alum Rock, CA
**nc73799186**	37.311	-121.675	8.1	3.5	2022-10-25 22:08:37	M 3.5 - 15km E of Seven Trees, CA
**nc73987616**	36.421	-121.927	7.1	2.9	2024-01-11 0:46:52	M 2.9 - 13 km N of Point Sur, CA
**nc73797331**	36.913	-122.160	8.1	2.4	2022-10-21 14:29:11	M 2.4 - 13km WSW of Santa Cruz, CA
**nc75153077**	36.838	-122.129	10.5	2.3	2025-03-22 5:26:54	M 2.3 - 17 km SW of Santa Cruz, CA
**nc73974081**	36.603	-122.046	7.5	2.3	2023-12-10 7:39:08	M 2.3 - 9 km WNW of Del Monte Forest, CA
**nc75043822**	36.916	-122.169	10.5	2.1	2024-08-05 14:28:43	M 2.1 - 14 km WSW of Santa Cruz, CA
**nc73804755**	36.991	-122.240	1.1	2.1	2022-11-13 19:16:32	M 2.1 - 10km SW of Bonny Doon, CA
**nc75058341**	36.697	-122.080	11.5	2.0	2024-09-07 0:22:54	M 2.0 - 17 km NW of Pacific Grove, CA
**nc73774875**	36.792	-121.912	10.9	2.0	2022-09-03 4:00:06	M 2.0 - 11km W of Moss Landing, CA

Figure S1: Table of replay events.

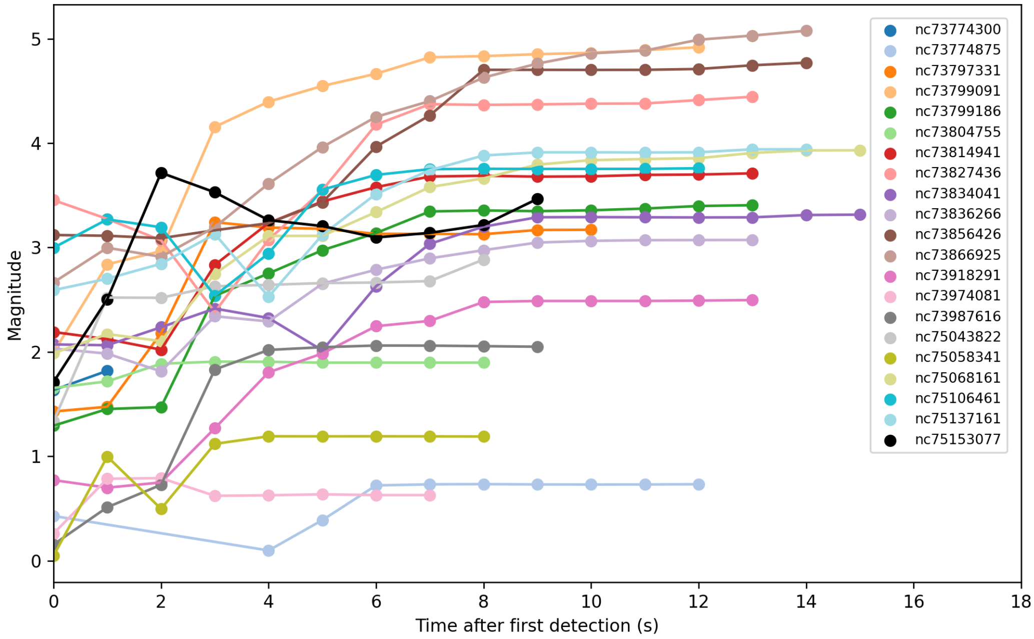

Figure S2: Magnitude estimation evolution for all replay events.

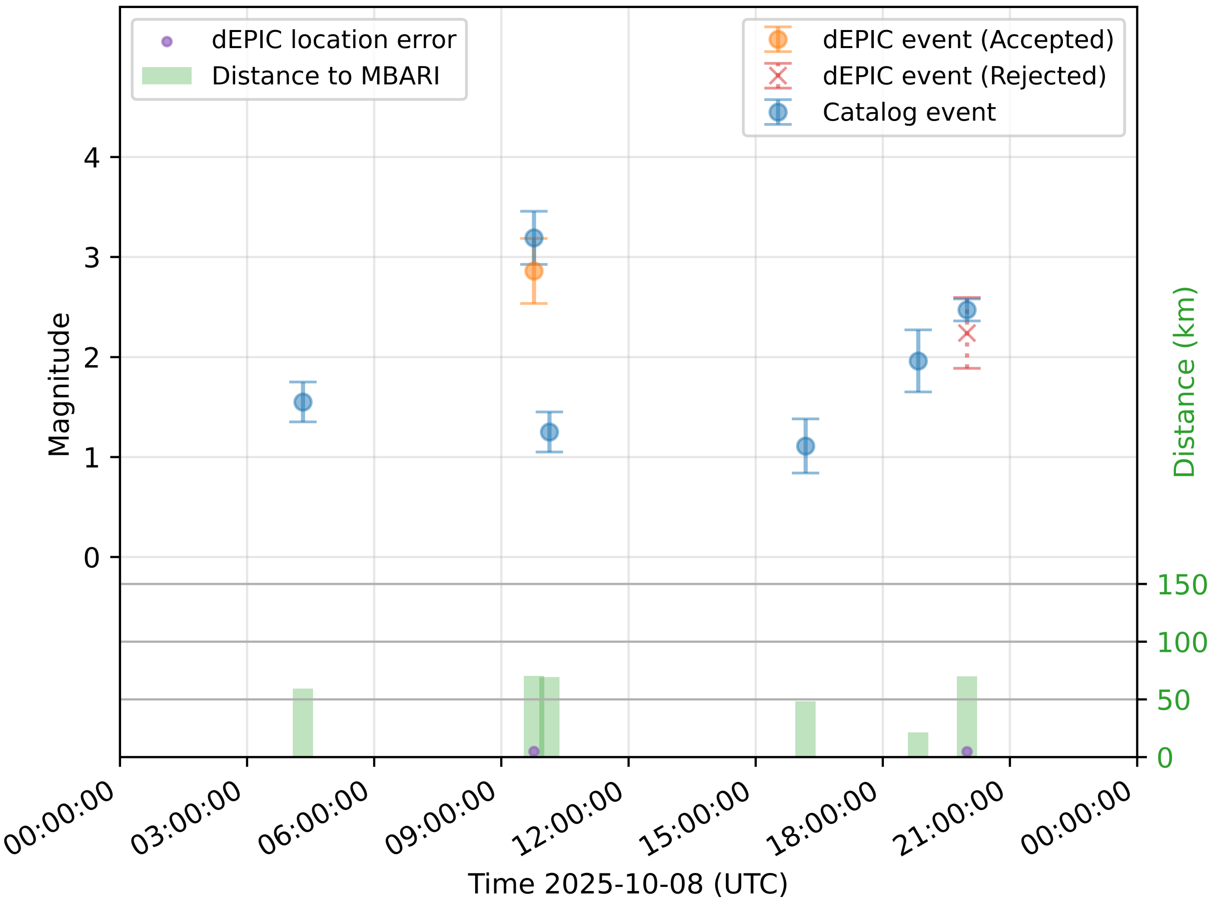

Figure S3: Full-day dEPIC real-time results for 08 October 2025. Accepted (orange circles) and rejected (red crosses) dEPIC solutions with magnitude estimates and median absolute deviation values. Catalog events (blue circles) are shown with magnitude uncertainties. Green bars indicate event distances from the DAS array. Solutions with maximum magnitude estimates associated with catalog events are shown with location errors (purple dots).

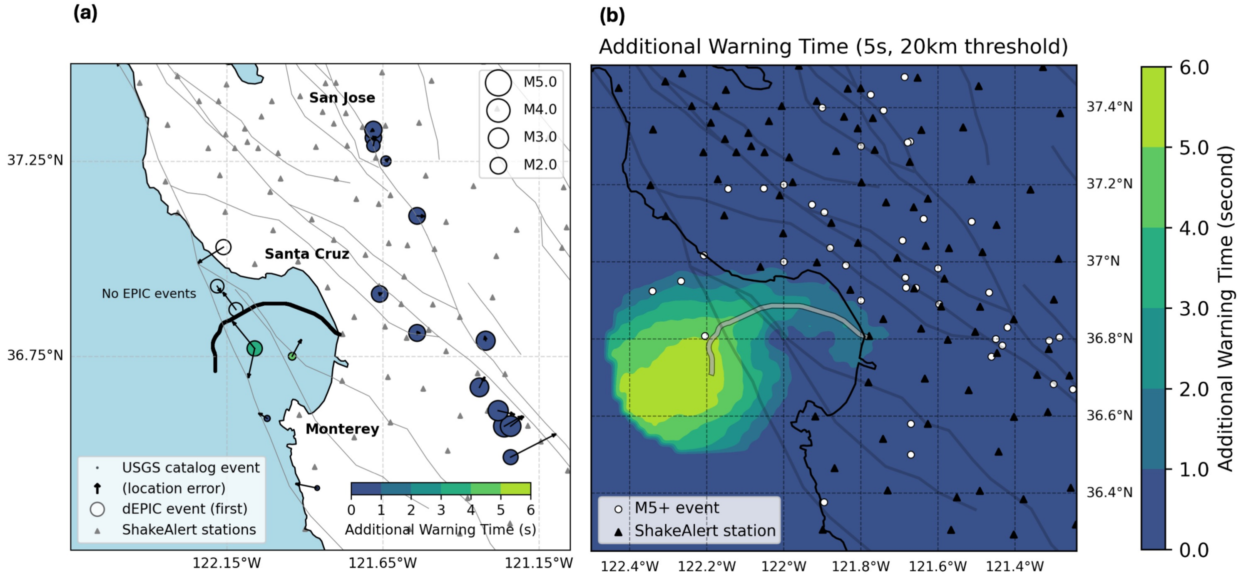

Figure S4: (a) Observed additional warning time from replays; (b) Estimated additional warning time as defined in Gou et al., 2025.

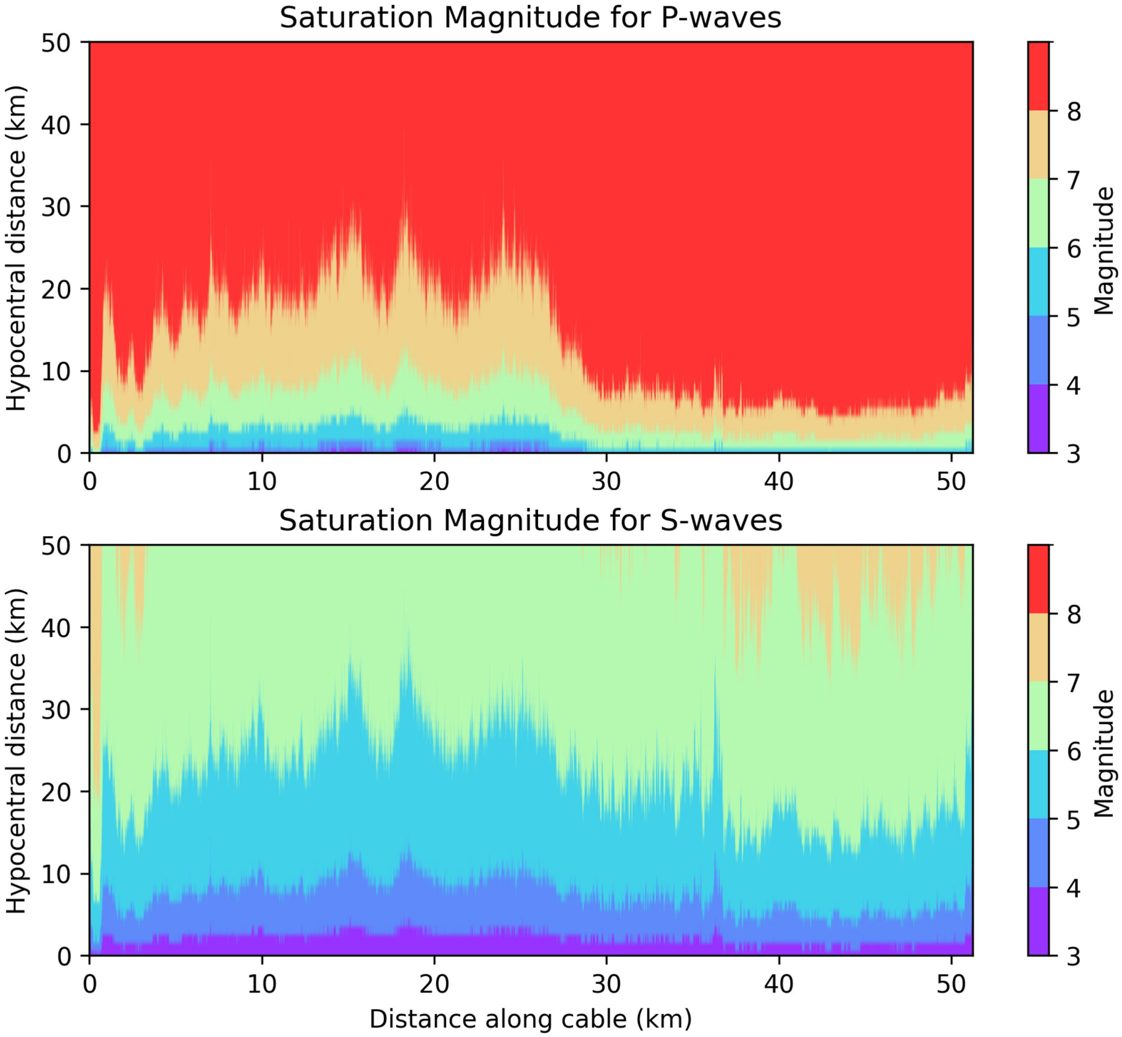

Figure S5: Saturation magnitude as a function of hypocentral distance and channel number, estimated from the theoretical maximum strain rate measurable with the SeaFOAM DAS array configuration. The upper panel shows results for P waves and the lower panel for S waves, based on the calibrated empirical magnitude – peak strain-rate relationship in Gou et al., 2025. The estimation is based on a configuration with a 20.4-m gauge length and a 200-Hz repetition rate.

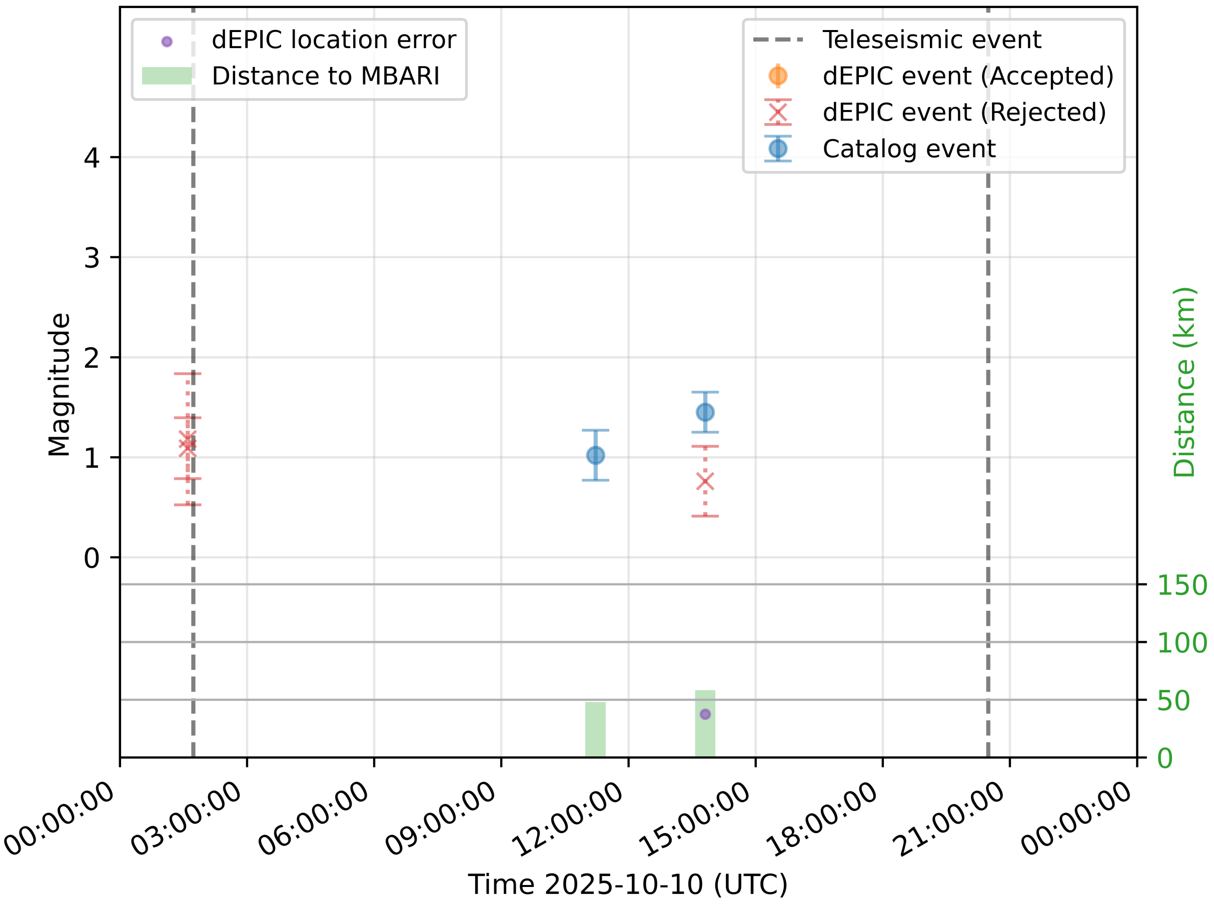

Figure S6: Full-day dEPIC real-time results for 10 October 2025. Same legend as in Figure S3, with black dashed lines indicating two M7+ teleseismic events.

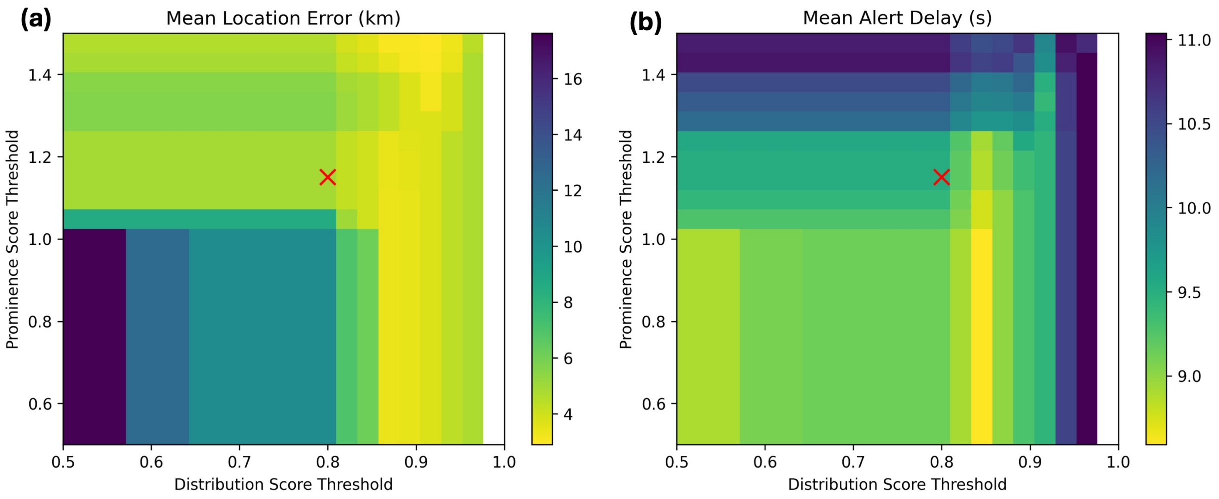

Figure S7: Evaluation of different thresholds for the two location metrics to assess the trade-off between alert timeliness and location accuracy. The adopted thresholds (prominence = 1.15 and distribution = 0.80, shown as red crosses) achieve a balanced performance and have proven effective in real-time tests.

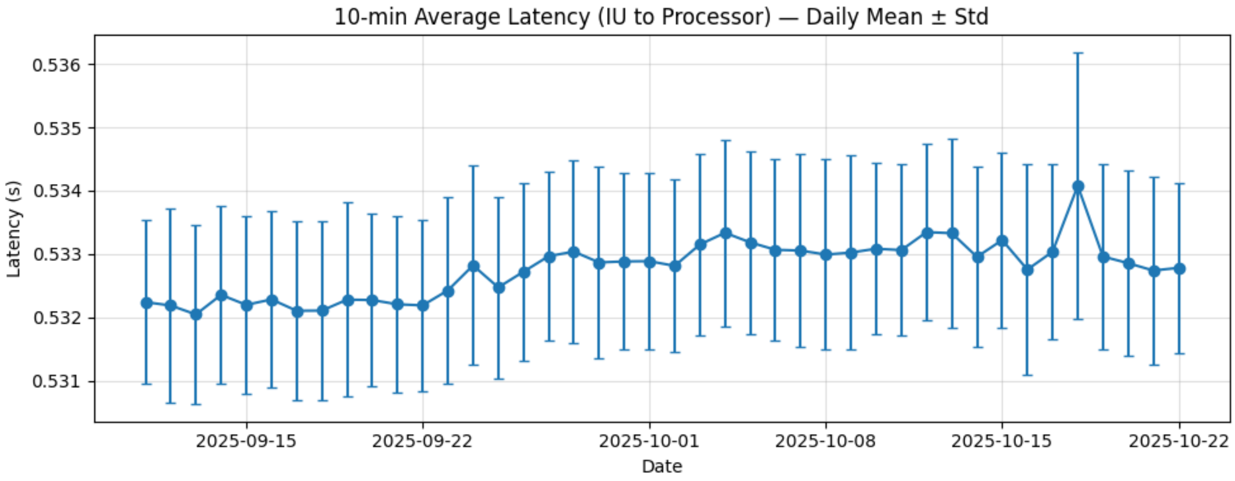

Figure S8: Daily 10-min average latency data from real-time operation. The average latency between DAS interrogator and the dEPIC processor is around 0.53s.

1 REFERENCES

1. Gou, Y., Allen, R. M., Zhu, W., Taira, T. and Chen, L.-W. Leveraging submarine DAS arrays for offshore earthquake early warning: A case study in monterey bay, California. Bull. Seismol. Soc. Am. 115, 516–532 (2025).
